# Salidroside suppresses solar ultraviolet-induced skin inflammation by targeting cyclooxygenase-2

**DOI:** 10.18632/oncotarget.8300

**Published:** 2016-03-23

**Authors:** Dan Wu, Ping Yuan, Changshu Ke, Hua Xiong, Jingwen Chen, Jinguang Guo, Mingmin Lu, Yanyan Ding, Xiaoming Fan, Qiuhong Duan, Fei Shi, Feng Zhu

**Affiliations:** ^1^ Department of Dermatology of the General Hospital of Air Force, Beijing, 100142, PR China; ^2^ Department of Biochemistry and Molecular Biology, Tongji Medical College, Huazhong University of Science and Technology, Wuhan, Hubei, 430030, PR China; ^3^ Department of Pathology, Tongji Hospital, Huazhong University of Science and Technology, Wuhan, Hubei, 430030, PR China

**Keywords:** salidroside, cyclooxygenase-2, solar UV, skin inflammation, p38

## Abstract

Solar ultraviolet (SUV) irradiation causes skin disorders such as inflammation, photoaging, and carcinogenesis. Cyclooxygenase-2 (COX-2) plays a key role in SUV-induced skin inflammation, and targeting COX-2 may be a strategy to prevent skin disorders. In this study, we found that the expression of COX-2, phosphorylation of p38 or JNKs were increased in human solar dermatitis tissues and SUV-irradiated human skin keratinocyte HaCaT cells and mouse epidermal JB6 Cl41 cells. Knocking down COX-2 inhibited the production of prostaglandin E_2_ (PGE_2_), the phosphorylation of p38 or JNKs in SUV-irradiated cells, which indicated that COX-2 is not only the key enzyme for PGs synthesis, but also an upstream regulator of p38 or JNKs after SUV irradiation. The virtual ligand screening assay was used to search for natural drugs in the Chinese Medicine Database, and indicated that salidroside might be a COX-2 inhibitor. Molecule modeling indicated that salidroside can directly bind with COX-2, which was proved by *in vitro* pull-down binding assay. *Ex vivo* studies showed that salidroside has no toxicity to cells, and inhibits the production of PGE_2_, phosphorylation of p38 or JNKs, and secretion of interleukin-6 (IL-6) and tumor necrosis factor-alpha (TNF-α) caused by SUV irradiation. *In vivo* studies demonstrated that salidroside attenuates the skin inflammation induced by SUV. In brief, our data provided the evidences for the protective role of salidroside against SUV-induced inflammation by targeting COX-2, and salidroside might be a promising drug for the treatment of SUV-induced skin inflammation.

## INTRODUCTION

Excessive exposure to solar UV (SUV) is associated with numerous human skin disorders, such as skin inflammation, photoaging, and carcinogenesis [1–3]. SUV comprises approximately 95% UVA and 5% UVB. Exposure to UV is known to induce clustering of some kinds of cell-surface receptors and to transducer intracellular signals [4]. UVA activates p38 and JNKs, while p38 is the primary signaling pathway activated by UVB [5, 6]. Consequently, these kinases activate their downstream transcription factors, such as nuclear factor-kappa B (NF-κB) and (AP-1). Finally, pro-inflammatory factors, such as cyclooxygenase 2 (COX-2), interleukins (IL-1, IL-6, et al), tumor necrosis factor-alpha (TNF-α), are produced. The skin inflammation also precedes photoaging and carcinogenesis. Thus anti-inflammation is an important strategy in SUV induced skin disorders.

Cyclooxygenase (COX) is the rate-limiting enzyme in the production of prostaglandins (PGs) from arachidonic acid (AA) [2]. There are two major isoforms of COX, COX-1 and COX-2. COX-1 is normally constitutively expressed, but COX-2 is inducible by many stimuli including SUV [7]. Increased level of COX-2 induced by SUV irradiation causes inflammation, cell proliferation, tumor promotion and angiogenesis [8, 9]. COX-2 inhibition prevents skin inflammation, aging and carcinogenesis, representing a potential strategy for preventing solar UV related skin disorders. Nonsteroidal anti-inflammatory drugs have been widely used to reduce PGs and COX-2 expression. Previous studies showed that COX-2 inhibitors such as celecoxib suppress UVB-induced skin inflammation and tumor formation [10]. A number of COX-2 inhibitors have been developed, but most of them are restricted due to the different risk factors [11–13]. For example, nonselective nonsteroidal anti-inflammatory drugs (ns-NSAIDs) inhibit both COX-1 and COX-2, which exhibit significant gastric-intestinal (GI) toxicity, such as development of gastric ulcers and gastrointestinal bleeding. Large clinical studies identified an increased risk of developing cardiovascular conditions, including myocardial infarction and stroke, with the use of selective COX-2 inhibitors. Therefore, it is necessary to identify novel and nontoxic COX-2 inhibitors.

Salidroside, p-hydroxyphenethyl-b-D-glucoside, a phenylpropanoid glycoside is one of the major components extracted from *Rhodiola rosea*, a plant with extensive history as a traditional medicine, particularly in cultures in the northern latitudes of Europe, Asia, and North America [14, 15]. In this study, we identified that salidroside is a novel COX-2 inhibitor by structure-based virtual screening assay, and it exerts potent anti-inflammatory effect on SUV-induced skin inflammation *ex vivo* and *in vivo.*

## RESULTS

### The expression of COX-2, phosphorylation of p38 or JNKs, Ki67 and CD45 are increased in human solar dermatitis

**Figure 1:**
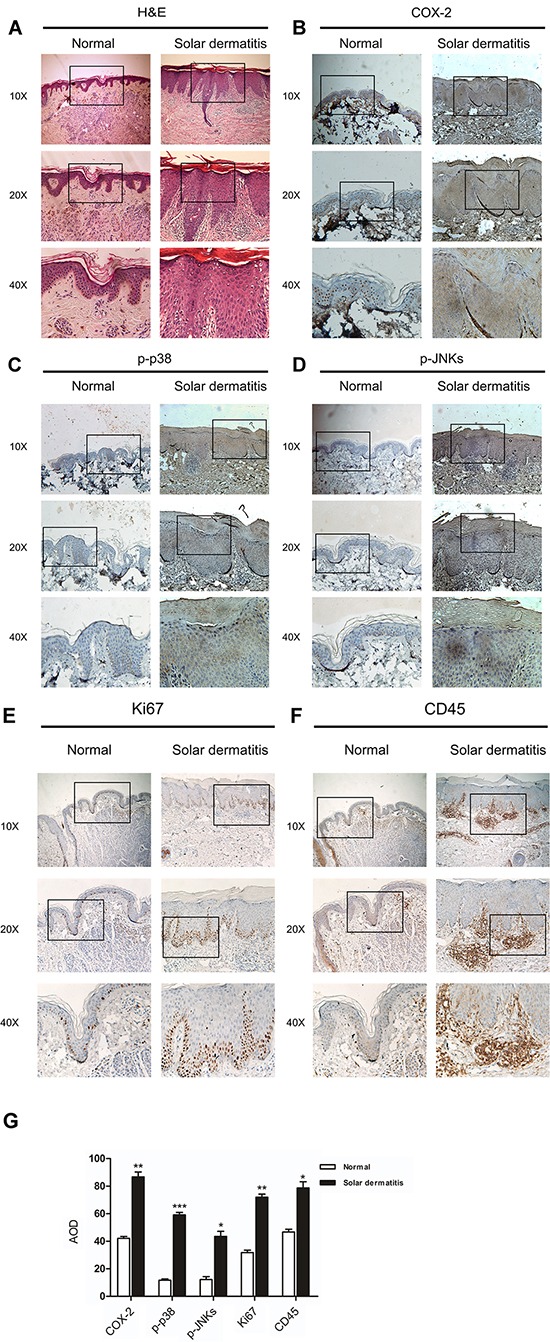
The levels of COX-2, phosphorylation of p38 or JNKs, Ki67 and CD45 are increased in human solar dermatitis **A.** The pathological features were shown in solar dermatitis compared with the normal skin from H&E staining. **B–F.** Immunohistochemistry analysis was used to determine the levels of COX-2, phospho-p38 or phospho-JNKs, Ki67 and CD45 in human solar dermatitis and normal skin tissues. **G.** Quantification of COX-2 expression, phosphorylation levels of P38, JNKs, Ki67 and CD45 were analyzed and data were shown as the average optical density (AOD). Data were presented as means ± SEM of values. Significant difference were indicated (*p<0.05; **p<0.01; ***P<0.001).

Previous studies have reported that COX-2 is closely associated with the SUV induced skin inflammation[8], p38 activation is the dominant SUV-induced signaling transduction pathway, and JNKs were activated by both UVA and UVB[5, 6]. The solar dermatitis, also known as sunburn, is caused by exposing in SUV. In order to know pathological changes in solar dermatitis, the cases of solar dermatitis and normal skin samples were detected. The pathological features were shown by H&E staining. Epidermal hyperkeratosis, hyperplasia of epidermis, furcella extension, the multifocal intercellular edema, dermal shallow vasodilation, priority with lymphocytes of inflammatory cells infiltration around the blood vessels were observed in solar dermatitis samples (Figure 1A). The skin inflammation caused by SUV always accompanied with elevated COX-2 and activation of cell signal pathways, such as p38, JNKs [17]. Ki67 was detected as cell proliferation marker, and CD45 was detected as an inflammation marker. The results indicated that the expression of COX-2, phosphorylation of p38 or JNKs, Ki67 and CD45 were remarkably increased in solar dermatitis (Figure 1B-1G).

### SUV irradiation induces the expression of COX-2, the phosphorylation of p38 or JNKs in HaCaT and JB6 Cl41 cells

**Figure 2:**
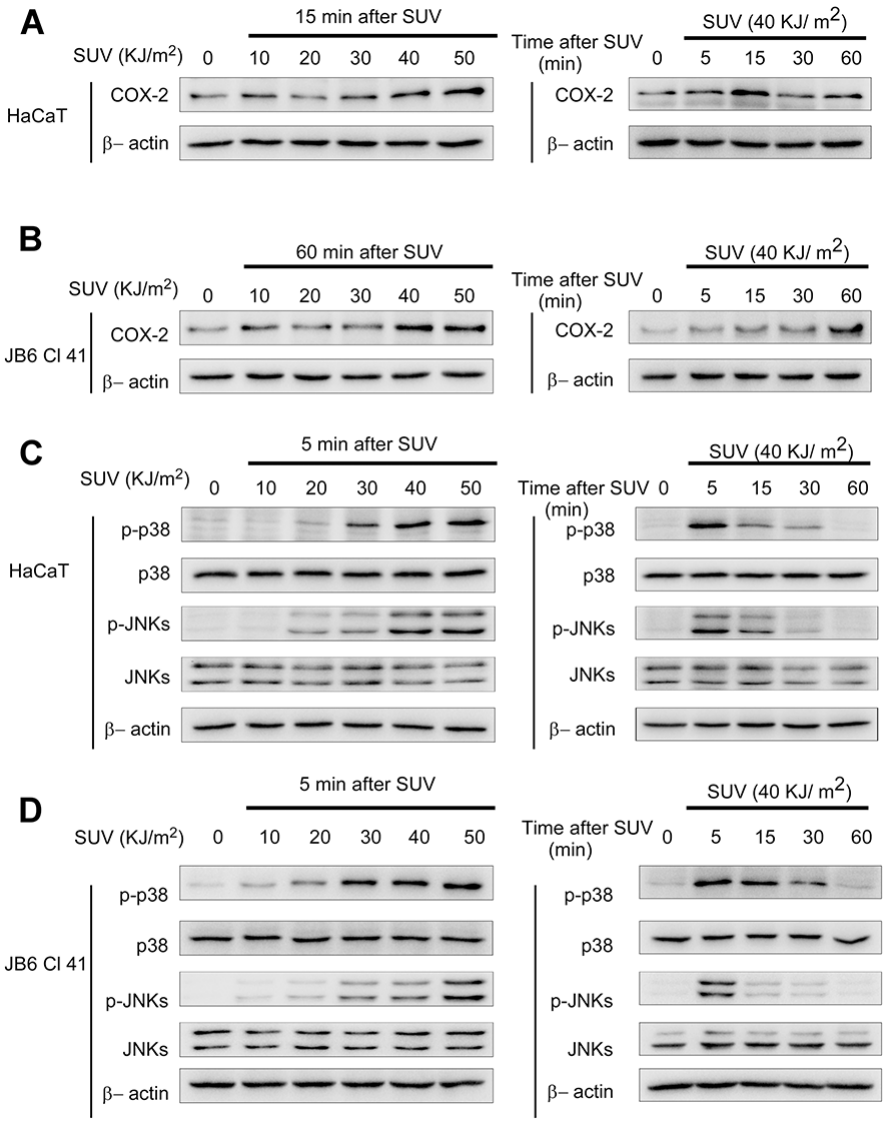
SUV irradiation induces COX-2 expression, the phosphorylation of p38 or JNKs in HaCaT and JB6 Cl41 cells **A** and **B.** COX-2 expression was increased by SUV irradiation in HaCaT and JB6 Cl41 cell models. The cell lysates (30 μg) were subjected to 10% SDS-PAGE. Protein bands were detected by Western blot according to Materials and Methods. **C** and **D.** Phosphorylation of p38 or JNKs in HaCaT or JB6 Cl41 cells was dramatically activated by SUV as indicated in a dose- or time-dependent manner. Data were representative of three independent experiments that gave similar results.

Human keratinocyte HaCaT cell line and Mouse epidermal JB6 Cl41 cell line were used as cellular models to research SUV induced skin inflammation [9]. The levels of COX-2, phosphorylated p38 or JNKs were examined after SUV irradiation with different doses or time points. The expression of COX-2 was increased at 15 min after 40 KJ /m^2^ SUV irradiation in HaCaT cells, or 60 min after similar SUV irradiation in JB6 Cl41 cells, respectively (Figure 2A, 2B). In both HaCaT and JB6 Cl41 cells, phosphorylated p38 or JNKs was increased by SUV irradiation in a dose-dependent manner, and the increasing effect was up to peak at 5 min after 40 KJ/m^2^ SUV irradiation (Figure 2C, 2D). These data indicated that SUV irradiation induced the expression of COX-2, the phosphorylation of p38 or JNKs in HaCaT or JB6 Cl41 cells, and 40 KJ/m^2^ SUV was determined for the following research.

### Knocking down COX-2 inhibits SUV-induced phosphorylation of p38 or JNKs in HaCaT cells

**Figure 3:**
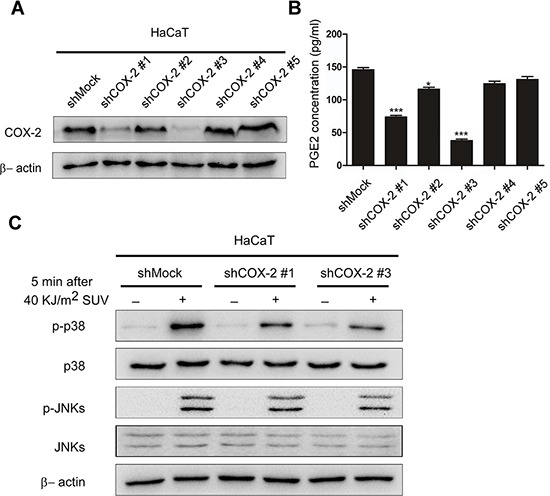
Knocking down COX-2 inhibites SUV-induced the phosphorylation of p38 or JNKs in HaCaT cells **A.** Expression of COX-2 in HaCaT cells was analyzed by Western blot. HaCaT cells were transfected with shMock or shCOX-2 (#1-#5), cell lysates were analyzed by western blot. **B.** Production of PGE_2_ in shMock or shCOX-2 (#1-#5) cells were measured by ELISA as described in Materials and Methods. COX-2 was dramatically decreased in shCOX-2 #1 and #3 cells, which were selected for further experiments. Data are presented as means ± SEM (n=3), *P < 0.05, ***P < 0.001, representing significant decrease compared with shMock cells. **C.** Knockdown of COX-2 inhibited SUV-induced phosphorylation of p38 or JNKs. HaCaT shMock cells and shCOX-2 #1 and #3 cells were stimulated with SUV (40 KJ/m^2^) and then harvested at 5 min. Proteins were analyzed by western blot. Data were representative of triplicate experiments.

COX-2 is closely related to SUV induced skin disorders [8, 9]. We detected the effect caused by COX-2 in SUV irradiated HaCaT cells by knocking down it. We set up shMock or shCOX-2(#1-#5) stable cell lines in HaCaT cells. Compared with control (shMock), the COX-2 expression and PGE_2_ production were decreased dramatically in shCOX-2 #1 and #3 cells (Figure 3A, 3B), which were used for the following study. The phosphorylation of p38 or JNKs was detected in cells at 5 min after SUV irradiation. Compared with shMock cells, the phosphorylated p38, or JNKs was remarkably decreased in COX-2 knocking down cells. In previous researches, COX-2 was regarded as the downstream effectors regulated by p38 or JNKs signal pathways [2, 9]. Our data suggested that COX-2 might be an upstream molecule for the activation of p38 or JNKs signal pathways in the response of SUV irradiation.

### Salidroside can directly bind with COX-2 and has no toxicity to HaCaT and JB6 Cl41 cells

**Figure 4:**
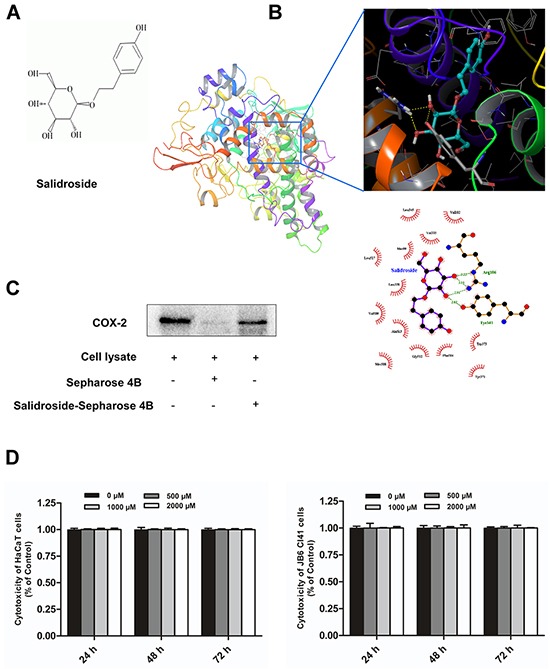
Salidroside can directly bind with COX-2 and has no toxicity to HaCaT and JB6 Cl41 cells **A.** The chemical structure of salidroside. **B.** Proposed molecular model of salidroside binding with COX-2. Salidroside binds to the active site of COX-2 by forming hydrogen bonds with protein amino acid residues ARG106 and TYR341. **C.** A pull-down assay was performed to detect the binding of salidroside with COX-2 *in vitro*. Lane 1 was input control (COX-2 protein standard); Lane 2 was the negative control, indicating no binding between COX-2 and Sepharose 4B beads; lane 3 indicated that COX-2 binds with Salidroside-Sepharose 4B beads. **D.** Salidroside had no cytotoxity on HaCaT and JB6 Cl41 cells. The cell viability was determined by MTS assay according to Materials and Methods. Data are shown as means ± standard deviation from triplicate experiments.

Traditional Chinese herbal medicines have been reported to have especial pharmacological effects, and always used for screening natural and nontoxic drugs. In this study, structure-based virtual screening was performed to identify novel COX-2 inhibitors in the Chinese Medicine Database. We identified salidroside (Figure 4A), isolated from *Rhodiola rosea*, as a potential COX-2 inhibitor based on its high docking score against COX-2. Molecule modeling indicated that the glycosyl of salidroside formed hydrogen bonds with COX-2 at amino acid residues ARG106 and TYR341 (Figure 4B). To validate the computational prediction, *in vitro* pull-down assay was performed, COX-2 was detected in sample eluted from sepharose 4B beads coupled with salidroside, but not in eluent from Sepharose 4B beads alone, which indicated salidroside could directly binds with COX-2 (Figure 4C).

To determine the cytotoxicity of salidroside, HaCaT and JB6 Cl41 cells were treated with various concentrations of salidroside (0, 500, 1000, 2000 μM), and measured by MTS assay at 24, 48, 72 h. The results indicated that salidroside had no significant cytotoxicity on HaCaT and JB6 Cl41 cells (Figure 4D). In summary, salidroside can bind with COX-2 and has no cytotoxicity to cells.

### Salidroside inhibits the activity of COX-2 and decreases the inflammatory process induced by SUV-irradiation

**Figure 5:**
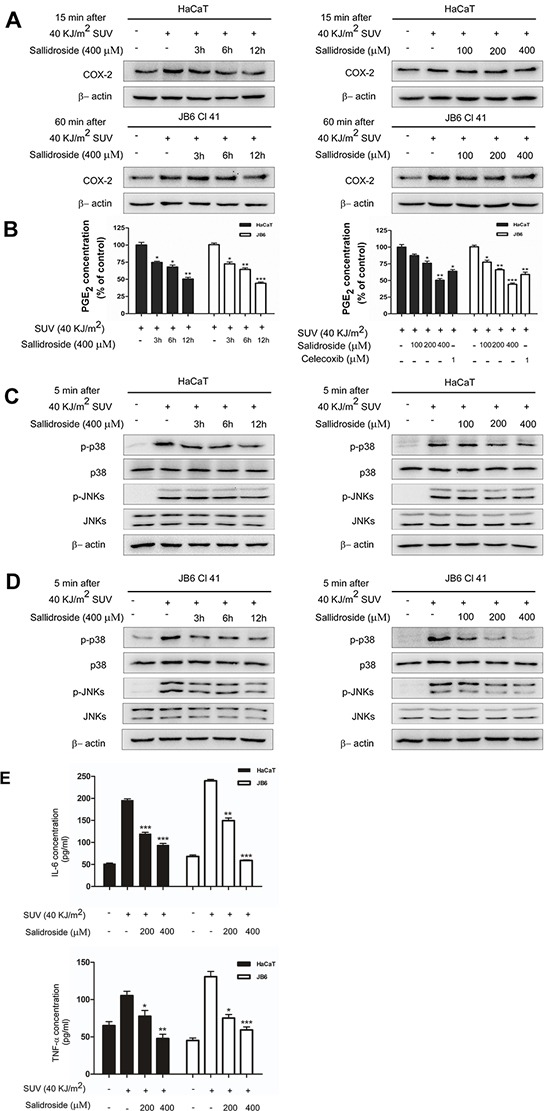
Salidroside inhibits the activity of COX-2, decreases the activation of p38 or JNKs signaling pathway and production of inflammatory factors in SUV-irradiated cells **A.** Salidroside inhibited the expression of COX-2 in a time-dependent manner, the obvious inhibition was observed at 12 h after SUV exposure. The cell lysates (30 μg) were subjected to 10% SDS-PAGE gel. **B.** Salidroside inhibited COX-2 activity and then markedly reduced PGE_2_ production in HaCaT and JB6 Cl41 cells. The PGE_2_ released in medium was measured by PGE_2_ assay kit according to the instructions described in Materials and Methods. **C** and **D.** Phosphorylation of p38 or JNKs was substantially attenuated in a dose- and time-dependent manner after SUV irradiation with salidroside treatment in HaCaT and JB6 Cl41 cells. Cells were pre-treated with salidroside and stimulated with SUV as indicated. The whole cell lysates were analyzed by western blot. **E.** Salidroside inhibited the secretion of IL-6 and TNF-α induced by SUV. Significant differences compared with the group treated with SUV alone (*P<0.05; **P<0.01; ***P<0.001).

Since salidroside directly binded with COX-2, next, we tested how it impacted on the intracellular COX-2. Firstly, the COX-2 level in salidroside pre-treated HaCaT cells or JB6 Cl41 cells was detected. The result showed that salidroside pre-treatment (400 μM) for 12 h decreased the COX-2 level at 15 min (for HaCaT cells) or 60 min (for JB6 Cl41 cells) after irradiated by 40 KJ/m^2^ SUV (Figure 5A), which indicated that salidroside could inhibit the SUV-induced COX-2 expression. Secondly, the production of PGE_2_ was detected to represent the activity of COX-2. Celecoxib, a well-known COX-2 inhibitor, was used as a positive control. The SUV irradiation-stimulated PGE_2_ production was decreased in both HaCaT cells and JB6 Cl41 cells by salidroside pre-treatment in a dose- and time-dependent manner (Figure 5B), the COX-2 specific inhibitor Celecoxib treatment showed the similar reducing effect. Thus, we next investigated the effect of salidroside on cell signals activated by SUV. The western blot results indicated that the phosphorylated of p38 or JNKs was substantially attenuated in dose- and time-dependent manners after pre-treatment of salidroside in HaCaT cells (Figure 5C) or JB6 Cl41 cells at 5 min after SUV irradiation (Figure 5D). The levels of COX-2 has not been changed at 5 min after SUV exposure, combined with the previous result that COX-2 might be an upstream regulator of p38 or JNKs, we concluded that salidroside inhibited the activity of COX-2 rather than changing its expression. To confirm whether salidroside suppressed the inflammation process, the secretion of IL-6 or TNF-α was measured in HaCaT and JB6 Cl41 cells. The result indicated that salidroside significantly inhibited the SUV induced IL-6 or TNF-α releasing from these cells (Figure 5E). Compared with effect on the expression of COX-2, salidroside showed more effective inhibition on the activity of COX-2. Overall, salidroside pre-treatment inhibited SUV-induced COX-2 activation, and in turn inhibited the activation of p38 or JNKs signaling pathway, and prevented the release of inflammatory factors such as IL-6 or TNF-α.

### Salidroside inhibits inflammation induced by SUV irradiation *in vivo*

**Figure 6:**
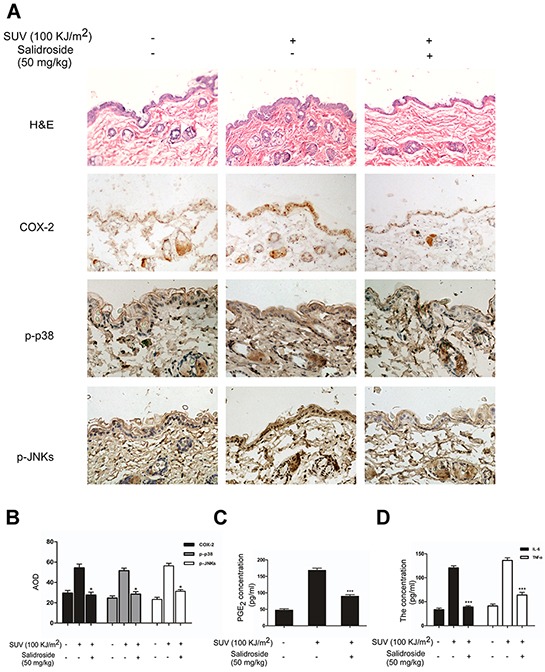
Salidroside inhibits inflammation induced by SUV irradiation *in vivo* **A.** Salidroside inhibited inflammation induced by SUV and down-regulated COX-2 level, phosphorylationof P38 and JNKs in mouse skin. Adult Babl/c mice were irradiated with one dose of solar UV light (100 KJ/m^2^) after pre-treatment of Salidroside or acetone in the dorsal skin for 3 h, and dorsal trunk skin samples for H&E staining and IHC were harvested at 24 h after irradiation. The levels of COX-2, phosphorylation of P38 or JNKs were shown mainly in epidermas of mice skin. **B.** Quantification of COX-2 expression, phosphorylation levels of p38, or JNKs were analyzed by the Medicine Image Analysis System (MIAS) and data are shown as the average optical density (AOD). Data were presented as means ± SEM of values from ten samples. The magnification of representative photos for H&E and the immunohistochemical staining was 20 ×. **C.** and **D.** The secretion of PGE_2_, IL-6 and TNF-α were significantly reduced by salidroside treatment in mouse skin tissues. Data were shown as mean ± SEM and asterisks indicated a significant inhibition by salidroside compared with the group treated with SUV alone (*P<0.05; ***P<0.001).

To further investigate anti-inflammatory activity of salidroside *in vivo*, it was used for treatment of SUV-induced skin inflammation in mouse model. The H&E staining results showed that the epidermal thickness increased significantly with the edema and epithelial cell proliferation, and infiltration of immunocytes in 100 KJ/m^2^ SUV irradiated mice (Figure 6A upper panel: middle column versus left column). While, epidermal thickness and inflammation caused by SUV irradiation were suppressed by 50 mg/kg salidroside treatment (Figure 6A upper panel: right column versus middle column). Since salidroside inhibited the activation of p38 or JNKs and production of inflammatory factors *ex vivo*, the levels of COX-2, phosphorylated of p38 or JNKs were detected by IHC, and the data indicated that SUV irradiation dramatically increased the levels of COX-2, phosphorylated p38 or JNKs, and salidroside treatment significantly reduced these effects caused by SUV (Figure 6A, 6B). Next, we tested the effect of salidroside on PGE_2_, IL-6 or TNF-α produced in mouse skin tissues. The production of these factors was remarkably increased in SUV irradiation group, and the increasing effect was significantly reduced by salidroside treatment (Figure 6B, C). Taken together, our findings provided evidences that salidroside suppressed skin inflammation by inhibiting COX-2 *in vivo*.

## DISCUSSION

SUV exposure leads to skin inflammation, which precedes photoaging and carcinogenesis [3]. UVA can promote photoaging and wrinkling of the skin and have potent carcinogenic effect [18], While UVB is the major risk factor for skin diseases including melanoma and nonmelanoma skin cancers, it can trigger the initiation, promotion, and progression phases of skin cancer [19, 20]. Both UVA and UVB causes skin inflammation by activating intracellular signal pathways. COX-2 is induced by p38 or JNKs signaling pathways after SUV irradiation, and catalyzes the synthesis of prostaglandins which not only promotes the process of inflammation, but also participate the carcinogenesis of skin [21–23]. So that COX-2 becomes a therapeutic target of interest for skin disorders. There are a lot of COX-2 inhibitors used in clinical. However the side effects of these drugs has been reported [24–27]. For example, a well-known COX-2 specific inhibitor, celecoxib, has been reported for multiple side effects, such as cardiovascular risk, acute kidney injury, and acute pancreatitis[24,28,29]. Beside the efficiency of such COX inhibitors, people are forced to pay more attention to the safety of COX-2 inhibitors.

In human solar dermatitis tissue and SUV-irradiated HaCaT and JB6 Cl41 cells, COX-2 is highly expressed accompanied with increasing of phosphorylated p38, or JNKs. After knocking down COX-2, not only the production of PGE_2_ in SUV-irradiated cells was decreased, but also the phosphorylated p38, or JNKs was attenuated, which indicated the crucial role of COX-2 in the inflammatory signals caused by SUV, and COX-2 might be an upstream activator of p38 or JNKs. COX-2 is regarded as an enzyme and its biological significance is mainly mediated by the production of prostaglandins [2]. These inflammatory lipids influence cellular physiology by different manners, which include (1) activation of G-protein coupled receptors, (2) inhibition of inflammation by activating corticosteroid-like receptors, (3) participation in receptor protein tyrosine kinase signal transduction [30]. These events might activate p38 or JNKs directly or indirectly. On the other hand, since the phosphorylated p38 or JNKs was detected as soon as 5 min after SUV exposure, the intracellular PGs has not been changes remarkably, we considered that COX-2 might be a upstream signal transductor and regulate the activation of p38 or JNKs by itself. How does it regulate the activation of p38 or JNKs? It is still unknown and needs to be proved by supportive experimental evidences.

Since the side effects of existing COX-2 inhibitors, it is necessary to develop new drugs targeting COX-2. Traditional Chinese herbal medicines have great therapeutic effects and pharmaceutical values, and gain more and more interests to search for natural drugs with high efficacy and low toxicity. Salidroside, a natural compound extracted *Rhodiola rosea L,* which has long been used as a traditional Tibetan medicine to relieve altitude sickness, other effects on neuro-protection, cardio-protection, anti-depression, anti-fatigue, and anti-tumor have been reported [14, 15]. Salidroside is thought to be one of the most effective compounds from *Rhodiola rosea L* for its multiple functions, such as anti-aging, anti-cancer, anti-inflammation, anti-oxidative stress, hepatoprotective properties and neuroprotective effect [31–36]. But the specific molecular target of salidroside has not been reported. In this study, structure-based virtual ligand screening was performed to screen a selective COX-2 inhibitor from Chinese Medicine Database and identified salidroside might be a COX-2 inhibitor. Molecule modeling indicated that the glycosyl of salidroside forms hydrogen bonds with COX-2, *in vitro* pull-down assay was performed and indicated that cellular derived COX-2 binds with salidroside directly. The MTS study determined that salidorside has no cytotoxicity even the concentration up to 2000 μM and the incubation time up to 72 h, which indicated that the safety of salidroside for further utilization in therapy.

In order to study the effect of salidroside on COX-2, we detected how the expression and activity of COX-2 in SUV-induced skin inflammation are regulated by salidroside. In *ex vivo* studies, salidroside pretreatment reduced the SUV-induced COX-2 expression in a time-dependent manner, the obvious inhibition was observed at 12 h after SUV exposure in HaCaT cells. But the PGE_2_ production was reduced as early as 3 h after SUV exposure in both HaCaT cells and JB6 C141 cells. In SUV-induced inflammation, the production of PGE_2_ is increased earlier than the expression of COX-2, because of the activation of existing COX-2 by SUV irradiation. We also found that salidroside pretreatment significantly inhibited the phosphorylated p38 or JNKs after SUV exposure only for 5 minutes. In that condition, the expression of COX-2 had not be changed. So we determined that salidroside exerts its anti-inflammation role by inhibiting the activity of COX-2 rather than its expression, and the expression of COX-2 was reduced by salidroside due to the attenuated p38 or JNKs signals, like the reduced production of IL-6 and TNF-α. The *in vivo* study also proved that salidroside protects skin against the SUV-induced inflammation.

Our study indicated that salidroside inhibited SUV-induced skin inflammation by directly targeting COX-2 *in vitro* and *in vivo.* And we found that COX-2 is not only the key enzyme for the production of prostaglandins, but also works as an upstream regulator for p38 or JNKs. However, the interaction between COX-2 and p38 or JNKs is still unclear. It is worth noting that salidroside is a naturally occurring compound with high safety evidenced by the experimental data [37]. Besides the multiple effects, due to the good water solubility and low molecular weight (MW: 300.3), salidroside is well speculated that the compound may penetrate easily from the epidermis to the dermis with a proper preparation [15]. Overall, Salidroside showed great potential anti-inflammatory effect and might be a promising drug for the treatment of SUV-induced skin inflammation.

## MATERIALS AND METHODS

### SUV, reagents and antibodies

The SUV resource was purchased from Q-Lab Corporation (Cleveland, OH). The percentage of UVA and UVB of SUV lamps was measured by a UV meter and was 92.5% and 7.5% respectively. The dose of SUV was substituted by UVA and UVB in the following description, and 40 KJ/m^2^ SUV included 40 KJ/m^2^ UVA and 3.2 KJ/m^2^ UVB. Salidroside (with the purity>99%) was purchased from Shanghai Biyi Chemical Science and Technology Co. Ltd (Shanghai, China). Eagle's minimum essential medium (MEM), Dulbecco's Modified Eagle medium (DMEM), Fetal bovine serum (FBS) were purchased from Gibco (USA). The primary antibodies for COX-2, phosphorylated p38(Thr180/Tyr182), total p38, phosphorylated JNKs (Thr183/Tyr185), total JNKs were purchased from Cell Signaling Technology (USA), the primary antibody for β-actin and horseradish peroxidase (HRP) conjugated secondary antibody were obtained from Santa Cruz (USA) and Earth Ox life sciences company (San Francisco US). The prostaglandin E_2_ (PGE_2_) EIA Kit was purchased from Cayman Chemical Company (USA), the IL-6 and TNF-α ELISA kit were purchased from Dakewe Biotech Co. Ltd (Beijing, China). All antibodies were used following the instructions of the respective manufacturers.

### Cell culture

The human skin keratinocyte HaCaT cell line and mouse epidermal JB6 Cl41 cell line were obtained from American Type Culture Collection (ATCC, USA), cultured following the procedures provided by ATCC and were used within 6 months of resuscitation. The HaCaT were cultured in DMEM supplemented with 10 % FBS. JB6 Cl41 cells were cultured in MEM supplemented with 5% FBS. All the cells were cultured 37°C in humidified atmosphere containing 5% CO_2_.

### MTS assay

To estimate cell viability, cells (1000/well) were seeded in 96-well plates for 24 h, then treated with various concentrations of salidroside (0, 500, 1000, 2000 μM) for additional 24, 48, or 72 h. After incubation for various times, cell viability was measured by an MTS assay kit (Promega, Madison, WI) according to the manufacturer's instructions.

### Docking & homology modeling

Chinese Medicine Database was used in the virtual ligand screening, and salidroside was identified as a novel COX-2 inhibitor based on its docking score against COX-2. In order to explore the accurate binding model for the salidroside binding pocket of COX-2, we carried out molecular docking analysis using the AutoDock Vina software based on the modeling protein structure. Homology modeling on COX-2 was performed by Swiss-Model. Chain A of 3NT1 (PDB ID) was selected as the template protein from the PDB database. Prior to docking, a short protein structure optimization was added to the modeled receptor using AMBER ff99SB force filed. In docking, hydrogen atoms and charges of the receptor were added by using AutoDock Tools. The three-dimensional structure of salidroside was downloaded from ZINC database. The format of compound structure was converted to pdbqt format through Applied Chemistry Software Openbabel. Docking grid was proposed to enclose the substrate binding site of the protein. Finally, 9 docked poses of the ligand were produced for further analysis.

### *In vitro* pull-down binding assay

Salidroside-conjugated sepharose 4B beads or sepharose 4B beads were prepared as reported earlier [16]. For *in vitro* pull-down assay, cell lysates from HaCaT cells (1mg) were incubated with salidroside-Sepharose 4B or Sepharose 4B alone (as a control) in the reaction buffer [50 mM Tris-HCl (pH7.5), 5 mM EDTA, 150 mM NaCl, 1 mM dithiothreitol (DTT), 0.01% NP-40, 2 μg/ml bovine serum albumin, 0.02 mM phenylmethylsulfonyl fluoride (PMSF) and 1 μg/ml protease inhibitor cocktail (Roche)]. After incubation with gentle rocking at 4°C overnight, the beads were washed five times with washing buffer [50 mM Tris-HCl (pH7.5), 5 mM EDTA, 150 mM NaCl, 1 mM DTT, 0.01% NP-40, and 0.02 mM PMSF], and then the proteins bound to the beads were collected and analyzed by Western blot using COX-2 antibody.

### ELISA

HaCaT or JB6 Cl41 cells (2 × 10^5^) was plated in six-well dishes. When growing to 80% confluence, the cells were starved in serum-free medium for 12 h, then pre-treated with 400 μM salidroside for 3, 6, 12 h, or pre-treated with various concentrations of salidroside (0, 100, 200, 400 μM) or celecoxib (1 μM) for 12 h before exposed to 40 kJ/m^2^ SUV and further cultured for 18 h. Finally, the supernatant was collected for detection. PGE_2_ released into the medium was measured by the PGE_2_ EIA kit following the supplier's instructions. The released IL-6 or TNF-α in medium was determined by ELISA kit according to the manufacturer's instructions. PGE_2_, TNF-α, and IL-6 released in the mouse skin tissues were collected by homogenization and centrifugation, the supernatant was measured by EIA kit or ELISA kit as described above.

### Western blot

HaCaT cells (1 × 10^6^) or JB6 Cl41 (2 × 10^6^) were seeded in 10-cm dishes, cultured for 24 h, and starved for 12 h before SUV irradiation. For studying the effect of salidroside, the cells were pretreated with salidroside (400 μM) for further 3, 6, 12 h before SUV irradiation. After that the cells were harvested and disrupted in lysis buffer (150 mM NaCl, 1 mM EDTA, 1 mM EGTA, 10 mg/mL aprotinin, 10 mg/mL leupeptin, 5 mM phenylmethanesulfonyluoride (PMSF), 1 mM dithiolthreitol (DTT) containing 1% Triton X-100, pH7.4) followed by disruption by sonication and centrifugation at 12,000 rpm for 10 min. The quantity of protein was determined by the Bradford method. The samples with 5× SDS loading buffer were heated at 95°C for 10 minutes, and then cooled on ice. After that, the samples were subjected to 10% SDS-PAGE and transferred to a PVDF membrane (Millipore, Billerica, MA, USA). After blocking with 5% non-fat milk or 5% BSA, the membrane was incubated with a specific primary antibody at 4°C overnight. After incubated with HRP-conjugated secondary antibody, the protein bands were visualized by the ECL system (BIO-RAD, USA). All experiments were repeated at least three times.

### Animal study

Adult Balb/c mice (6-8 weeks old) were purchased from the Center for Disease Control and Prevention (Hubei, China) and kept on a 12 h light/dark cycle at a controlled temperature with free access to food and tap water. The mice were divided into three groups: vehicle group (n=10), vehicle/SUV group (n=10), 50mg/kg salidroside /SUV group (n=10). The mice were shaved 24 h before experiment. In the vehicle group, the dorsal skin of mice was smeared with acetone for 3 h. In the vehicle/SUV group, the dorsal skin of mice was smeared with acetone for 3 h and then exposed to 100 KJ/m^2^ SUV. In the 50 mg/kg salidroside /SUV group, 50 mg/kg salidroside in acetone was smeared to the dorsal skin for 3 h and mice were exposed to 100 KJ/m^2^ SUV. The mice were euthanized and dorsal trunk skin samples were harvested at 24 h after SUV irradiation. One-half of the samples were immediately fixed in 4% paraformaldehyde and for hematoxylin and eosin (H&E) staining and immunohistochemistry (IHC). The other samples were frozen and used for ELISA analysis. All animal studies were conducted according to the guidelines approved by the Laboratory Animal Center of Huazhong University of Science and Technology.

### Immunohistochemistry

The human and mouse skin tissue sections (5μm) were performed antigen retrieval by microwave after deparaffinization and rehydration for 10 min in sodium citrate buffer. Then the sections were treated with 3% H_2_O_2_ for 10 min and blocked with 5% goat serum for 1 h at room temperature, then were incubated at 4°C overnight with primary antibodies as follows: 1:100 anti COX-2 raised in rabbit, 1:200 anti p-p38 (Thr180/Tyr182) raised in rabbit (Cell Signaling Technology) and 1:200 anti p-JNKs (Thr183/Tyr185). Then the primary antibody staining, detection and amplification using a biotinylated-streptavidin-HRP and DAB system, hematoxylin counterstaining. Slides were dehydrated through gradient alcohols to xylene and coverslipped with Permount TM Mounting Medium. All the slides were analyzed by Medicine Image Analysis System (MIAS).

### Statistical analysis

All quantitative data are expressed as mean ± SEM as indicated. The *t* test or one-way ANOVA was used for statistical analysis. A probability of *P* < 0.05 was considered statistically significant (*P < 0.05, **P < 0.01, ***P < 0.001).
